# Complex pancreaticobiliary maljunction diagnosed by endoscopic ultrasound: A case report

**DOI:** 10.1097/MD.0000000000040841

**Published:** 2024-12-13

**Authors:** Wen Xu, Yang Lv, Ying Zhu, Yingchun Zhang, Wei Gong, Xiaobing Cui

**Affiliations:** a Department of Gastroenterology, Shenzhen Hospital of Southern Medical University, Shen Zhen, Guangdong, China.

**Keywords:** choledochal cyst, chronic pancreatitis, endoscopic retrograde cholangiopancreatography, pancreatic divisum, pancreaticobiliary maljunction

## Abstract

**Rationale:**

This case report aims to enhance understanding of pancreatobiliary maljunction (PBM) and promote more proactive treatment.

**Patient concerns:**

The patient, a 24-year-old Chinese female, was admitted to the hospital on April 7, 2020, due to “recurrent abdominal pain for over 2 years, with a recent episode accompanied by nausea and vomiting for 1 day.” She had a previous history of gallstones.

**Diagnoses:**

The initial diagnosis upon admission was biliary acute pancreatitis. During the emergency endoscopic retrograde cholangiopancreatography (ERCP) procedure, anatomical abnormalities were discovered. Intraoperative endoscopic ultrasonography led to a diagnosis of complex PBM (JSPBM, type D) + choledochal cyst (Todani, Ic) + incomplete pancreatic divisum + early chronic pancreatitis. These diagnoses were confirmed by postoperative magnetic resonance cholangiopancreatography.

**Interventions:**

After multiple conservative treatments such as ERCP with accessory pancreatic duct stent placement, the patient underwent surgical treatment in April 2021, which included “laparoscopic left hemihepatectomy + choledochal cyst excision + cholecystectomy + hepatic portal cholangioplasty.”

**Outcomes:**

The patient has not experienced any abdominal pain since the surgery and is currently under regular follow-up.

**Lessons:**

Endoscopic ultrasound is effective for the diagnoses of complex PBM and incomplete pancreatic divisum. ERCP with pancreatic duct stent placement and surgical procedure is reliable for relieving the patient’s symptoms.

## 1. Introduction

Pancreaticobiliary maljunction (PBM) refers to a congenital developmental malformation where the pancreatic duct and bile duct abnormally join outside the wall of the duodenum in anatomical terms. Due to the inability of the sphincter of Oddi to affect the confluence site, there is mixing and reflux of pancreatic juice and bile, which can ultimately lead to various pathological changes in the biliary tract and pancreas.^[1]^ PBM is commonly seen in children under 10 years old, with a higher incidence in females, and is primarily of the bile duct dilation type. In adult patients, it is often manifested as non-dilated bile duct type PBM.^[2]^ This article presents a case of a young female patient diagnosed with complex PBM through endoscopic ultrasonography, and discusses the long-term complications affecting both the biliary tract and pancreas. The aim is to enhance understanding of this disease and promote more proactive treatment.

## 2. Clinical data

### 2.1. General Information

The patient is a 24-year-old female who was admitted to the hospital on April 7, 2020, due to “recurrent abdominal pain for over 2 years, with a recent episode accompanied by nausea and vomiting for 1 day.” One day prior to admission, the patient experienced episodic cramping pain in the upper abdomen, accompanied by nausea and vomiting of gastric contents once. Upon physical examination, tenderness in the right upper abdomen was noted, and Murphy sign was positive. Emergency blood routine test results showed: white blood cell 11.04 × 10^9^/L, neutrophil 8.04 × 10^9^/L. Abdominal ultrasound revealed stones in the common bile duct with proximal dilation of the common bile duct, and a rough and thickened gallbladder wall. She has a history of recurrent similar abdominal pain and a 2-year history of “gallstones,” with no history of smoking, alcohol consumption, or other unhealthy habits and no family history.

### 2.2. Examinations

**Figure 1.**
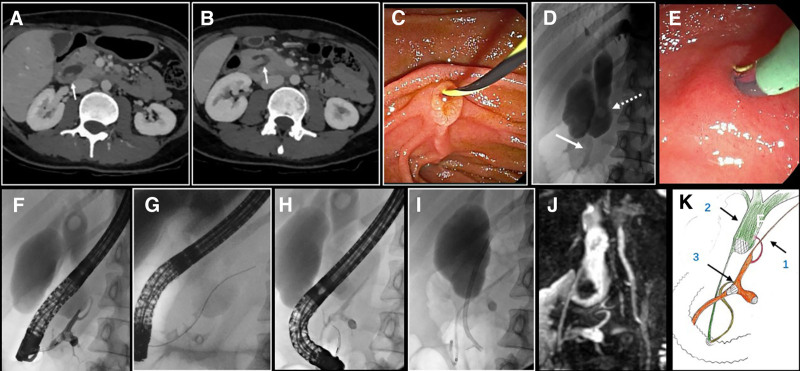
Upper abdominal CT, ERCP, MRCP, and diagnostic schematic from April 2020. (A) CT image showing a common bile duct stone (arrow). (B) CT image demonstrating dilation of the main pancreatic duct with an arcuate convergence of branch pancreatic ducts. (C) ERCP procedure with catheterization of the main papilla bile duct. (D) Cholangiogram revealing cystic dilation with filling defect in the middle-upper segment of the common bile duct (dashed arrow) and stenosis in the pancreatic segment (arrow). (E) ERCP procedure with catheterization of the accessory pancreatic duct. (F) Accessory pancreatic ductogram showing local dilation and filling defect. (G) Guidewire insertion into the accessory pancreatic duct. (H) After placement of a stent in the accessory pancreatic duct, catheterization of the main pancreatic duct becomes difficult. (I) Post-ERCP procedure with biliary stent placement. (J) Postoperative MRCP. (K) Diagnostic schematic illustration: (1) Pancreatic duct branch communicating with the common bile duct, (2) Dilated common bile duct with stones, and (3) Pancreatic divisum, with local dilation and protein plugs in the accessory pancreatic duct. ERCP = endoscopic retrograde cholangiopancreatography, MRCP = magnetic resonance cholangiopancreatography.

Laboratory tests: high sensitive C-reaction protein 93.99 mg/L, lipase 7055.31 U/L, amylase 545.37 U/L, carbohydrate antigen 199 79.84 U/mL. The remainder of the liver function, kidney function, troponin, electrolytes, rheumatism indicators, immunoglobulins, immunoglobulin G4, carcino-embryonic antigen, alpha-fetoprotein, and blood lipid tests showed no significant abnormalities. Enhanced CT of the upper abdomen: Swelling of the pancreatic head with surrounding effusion, considering the possibility of pancreatitis; fatty infiltration in the body and tail of the pancreas; dilation of the main pancreatic duct in the pancreatic head with convergence of branch pancreatic ducts; stones in the common bile duct with dilation of intrahepatic and extrahepatic bile ducts (Fig. 1A and B).

Initial diagnosis: (1) Acute pancreatitis (mild, initial onset, biliary in origin); (2) Common bile duct stones with dilation of the common bile duct. Symptomatic and supportive treatment was administered according to acute biliary pancreatitis, and endoscopic retrograde cholangiopancreatography (ERCP) was planned for the removal of common bile duct stones.

On April 8, 2020, ERCP was performed. After cannulation of the major duodenal papilla and cholangiography (Fig. 1C), cystic dilation and filling defects were found in the middle and upper segments of the common bile duct, with a notably thin “pancreatic segment.” The stones could not be removed (Fig. 1D), and cannulation of the main pancreatic duct was difficult. To establish a definitive diagnosis, intraoperative endoscopic ultrasound (EUS) was arranged.

**Figure 2.**
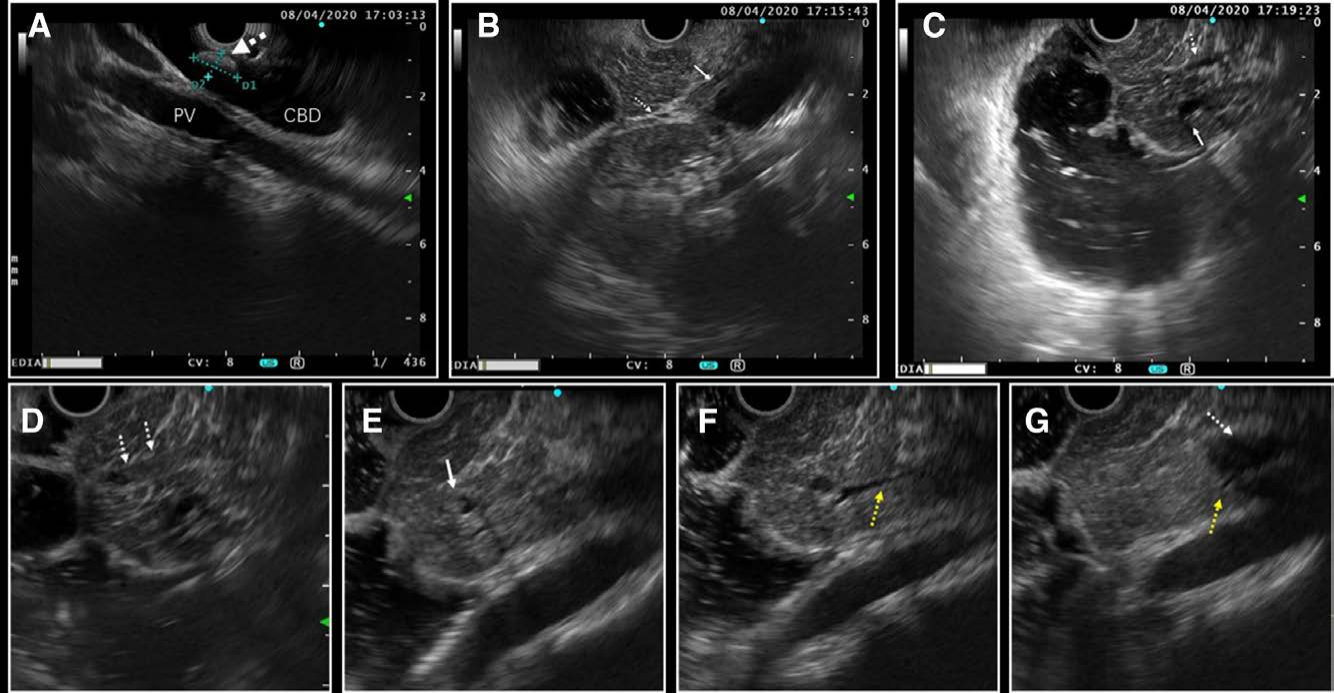
Intraoperative EUS examination during ERCP. (A) Sphincter of Oddi scan showing dilation of the common bile duct with a common bile duct stone (dashed arrow). (B) Duodenal bulb scan revealing dilation of the mid-segment of the common bile duct (arrow) and stenosis in the pancreatic segment (dashed arrow). (C) Local dilation of the main pancreatic duct (arrow) with visibility of the accessory pancreatic duct (dashed arrow). (D) Isoechoic material within the accessory pancreatic duct (dashed arrow). (E) Isoechoic material within the main pancreatic duct (arrow). (F) Branch pancreatic ducts (yellow dashed arrows). (G) Pancreatic duct branch continuous with the common bile duct (yellow dashed arrow), indicated by the white dashed arrow pointing to the common bile duct. ERCP = endoscopic retrograde cholangiopancreatography, EUS = endoscopic ultrasound.

EUS during ERCP on April 8, 2020: The middle and upper segments of the common bile duct were dilated with a diameter of 14.5mm, and the wall thickness was not significantly increased. A stone measuring 17.5 × 6.0 mm was present in the common bile duct (Fig. 2A). The pancreatic segment of the common bile duct was stenotic (Fig. 2B). The body and tail of the pancreas showed high echogenicity, while the head of the pancreas exhibited inhomogeneous low echogenicity with scattered striated high echogenicity (Fig. 2C). The accessory pancreatic duct was dilated with isoechoic contents within (Fig. 2D), and the main pancreatic duct was also dilated with isoechoic contents (Fig. 2E). The pancreatic duct branches were continuous with the common bile duct (Fig. 2F–G). The pancreatic ducts in the body and tail of the pancreas were not clearly visible.

Based on the intraoperative EUS scan results, it was considered that the patient had PBM and pancreatic duct protein plugs. After discussion, the procedures for bile duct dilation and fragmentation for common bile duct stone removal were canceled, and the ERCP procedure was continued: Cannulation via the minor papilla revealed that the accessory pancreatic duct drained the body and tail of the pancreas, with local dilation and filling defects (Fig. 1E–G). Cannulation of the main pancreatic duct was difficult, and imaging suggested the presence of thin communicating branches continuous with the dilated accessory pancreatic duct (Fig. 1H). After removing some protein plugs from the accessory pancreatic duct, a pancreatic duct stent and a biliary plastic stent were placed (Fig. 1I).

After the ERCP procedure, the patient’s abdominal pain significantly improved, and magnetic resonance cholangiopancreatography was performed to confirm the intraoperative diagnosis (Fig. 1J and K). Based on evidence-based medicine, further surgical treatment was recommended for the patient. However, the patient refused surgical treatment due to work and personal reasons and was discharged after recovery.

### 2.3. Diagnosis and differential diagnosis

Final Diagnosis: (1) Pancreatic anatomical abnormalities: PBM (PBMJ, Type D), choledochal cyst (Todani, Type Ic), incomplete pancreatic divisum; (2) Early chronic pancreatitis with multiple protein plugs in the pancreatic duct; and (3) Common bile duct stones. Specific diagnostic criteria are provided in the discussion section.

### 2.4. Treatment, follow-up, and outcome

On October 28, 2020, the patient visited Hospital A and attempted ERCP, but the bile duct stones were not successfully removed, and a 7fr biliary stent was placed.

**Figure 3.**
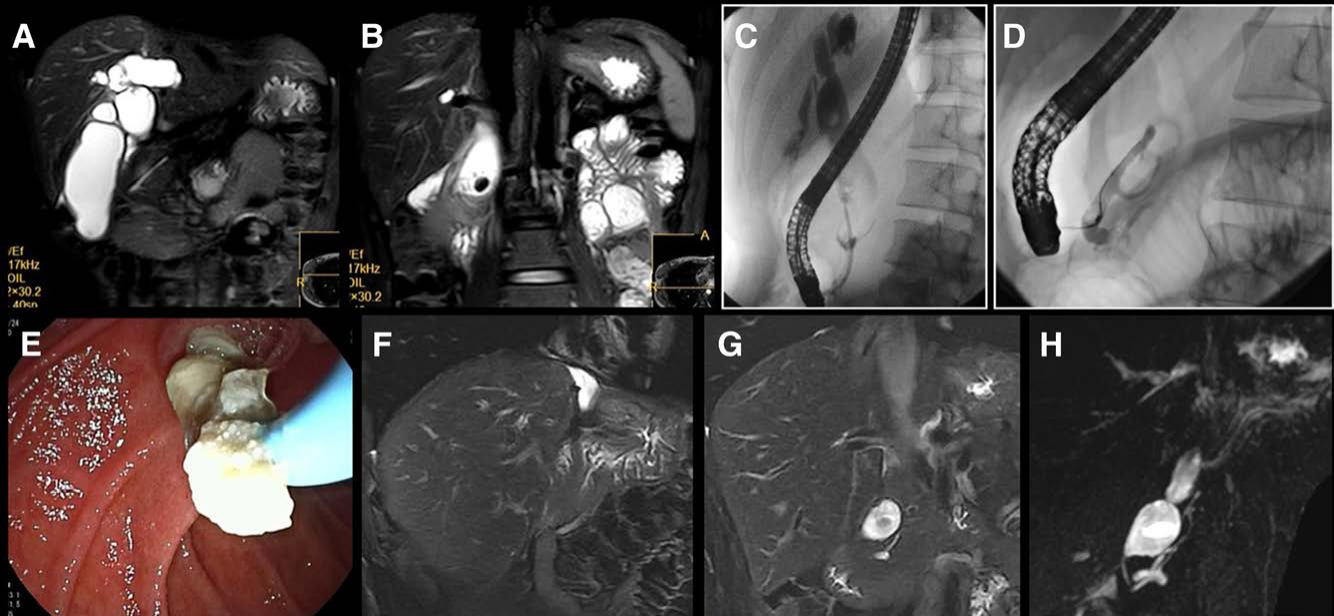
Treatment outcomes and follow-up process: (A) MRI in November 2020 showing dilation of the common bile duct and left hepatic duct. (B) MRI in November 2020 revealing the original common bile duct stone. (C) ERCP cholangiogram in November 2020. (D) ERCP accessory pancreatic ductogram in November 2020. (E) Partial removal of pancreatic duct stones during ERCP in November 2020. (F) MRI in April 2024 showing encapsulated effusion in the left lobe of the liver. (G) MRI in April 2024 revealing residual dilation and stones in the lower segment of the common bile duct. (H) MRCP in April 2024 showing no significant dilation or stones in the pancreatic duct. ERCP = endoscopic retrograde cholangiopancreatography, MRCP = magnetic resonance cholangiopancreatography.

November 17, 2020: The patient was readmitted to our hospital with dull pain in the right upper abdomen. MRI showed aggravated dilation of the common bile duct and dilation of the left hepatic duct, with displacement of the pancreatic duct stent (Fig. 3A and B). ERCP was performed to replace the biliary stent, remove some accessory pancreatic duct stones, and place an accessory pancreatic duct stent again (Fig. 3C–E).

On April 23, 2021, the patient visited Hospital B and underwent “laparoscopic left hemihepatectomy + cyst excision of the common bile duct + cholecystectomy + hepatic portal cholangioplasty + intraoperative choledochoscopy for stone removal + biliary stent removal.” No neoplastic changes were observed in the pathology report.

**Figure 4.**
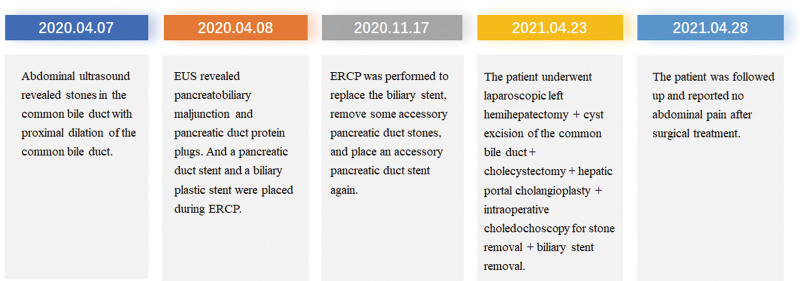
The timeline of the diagnosis and treatment process of this case.

On April 28, 2024, the patient was followed up in our outpatient department and reported no abdominal pain after surgical treatment, with regular follow-up at another hospital. MRI + magnetic resonance cholangiopancreatography of the upper abdomen indicated encapsulated effusion in the left hepatic lobe region, residual cyst with stones in the pancreatic segment of the common bile duct, pancreatic divisum, and no dilation or stones in the original pancreatic duct (Fig. 3F–H). The timeline of the diagnosis and treatment process is shown in Figure 4.

## 3. Discussion

Pancreas divisum (PD) is caused by the failure of the fusion of the main and accessory pancreatic ducts during embryonic development, with an incidence rate of about 10%.^[3]^ PD can be classified into 4 types: (1) Complete PD, where the ventral and dorsal pancreatic ducts are completely separated, accounting for approximately 70% of all PD cases; (2) Incomplete PD: There are small communicating branches between the ventral and dorsal pancreatic ducts, but they are not sufficient for adequate drainage of pancreatic juice, accounting for approximately 15% of cases; (3) Absence of either the ventral or dorsal pancreatic duct, with the absence of the ventral pancreatic duct being more common; and (4) Other types: Including Ansa pancreas and reverse PD, among others.^[4,5]^ In this patient, the accessory pancreatic duct primarily drains the body and tail of the pancreas, and there are delicate communicating branches with the main pancreatic duct, suggesting the presence of incomplete PD.

The classification criteria for PBM have continued to evolve over the years: (1) In 1977, Komi et al proposed a 3-type classification based on the anatomical angle of the pancreatic duct and bile duct junction: Type I, known as B-P type (choledochus joining the pancreatic duct, right-angle type); Type II, P-B type (pancreatic duct joining the choledochus, acute-angle type); and Type III, encompassing other complex types.^[6]^ (2) In 1992, the new Komi classification further subdivided Types I and II based on the presence (a) or absence (b) of dilation of the common channel, and refined Type III by incorporating Warshaw classification of pancreatic divisum^[7]^: Type IIIa (complete pancreatic divisum), Type IIIb (absence of the ventral pancreatic duct), and Type IIIc (incomplete pancreatic divisum). Within Type IIIc, further subdivisions were made based on the diameter of the pancreatic duct communicating branches: Type IIIc1 (slender communicating branches), Type IIIc2 (communicating branches equivalent to the pancreatic duct), and Type IIIc3 (dilated communicating branches or pancreatic duct). This standard is currently widely used.^[8]^ (3) In 2015, the Japanese Study Group on PBM proposed a new, simplified classification system due to the complexity of previous classifications: Type A (stricture type, with choledochal dilation and distal stenosis), Type B (non-stricture type, without distal choledochal stenosis), Type C (dilated common channel type, with distal choledochal stenosis and dilated common channel), and Type D (complex type, with additional abnormalities such as annular pancreas or pancreatic divisum).^[9]^ In this patient, ERCP and EUS revealed that the main pancreatic duct branch joined the common bile duct outside the duodenal wall, along with the presence of incomplete pancreatic divisum. Based on the above diagnostic criteria, the diagnosis is consistent with complex PBM (Japanese Study Group on PBM, Type D; Komi 1992, IIIc1).

Recurrent acute pancreatitis is defined as having at least 2 episodes of acute pancreatitis with no abnormal changes in pancreatic tissue or function during remission periods. In contrast, chronic pancreatitis (CP) is considered a progressive chronic inflammatory disease, and most previous diagnostic criteria were established based on late-stage classic cases.^[10,11]^ According to the 2009 Japanese Guidelines for Chronic Pancreatitis (revised in 2015) introduced the concept of early CP,^[12,13]^ and relevant international consensus was discussed in 2018,^[14]^ combined with the 2 minor diagnostic criteria of recurrent epigastric pain and elevated amylase levels, a diagnosis of early CP or CP (Clinical Stage 1) can be considered.

Biliary cysts, also known as biliary dilatation, are primarily caused by genetic factors and PBM, among other factors. Common complications include biliary stones, pancreatitis, and biliary tract cancer, with an overall canceration rate ranging from 2.5% to 30.0%. Surgical intervention is recommended as soon as possible upon diagnosis.^[15]^ There are various classification systems for biliary cysts: (1) The Todani classification system from 1977 is currently the most widely used: Type Ia: cystic dilation of the common bile duct; Type Ib: localized dilation of the common bile duct; Type Ic: diffuse fusiform dilation of extrahepatic bile ducts; Type II: diverticulum-like dilation of the common bile duct; Type III: intraduodenal dilation of the common bile duct, also known as choledochal cyst at the terminal end; Type IVa: multiple cystic dilations of intra- and extrahepatic bile ducts with intervening narrow rings; Type IVb: multiple cystic dilations of only extrahepatic bile ducts; Type V: single or multiple cystic dilations of intrahepatic bile ducts, known as Caroli disease.^[16]^ (2) Additionally, Davenport classification can also guide the treatment of biliary cysts.^[17]^ (3) In 2003, Todani et al found that Types Ia, Ic, and IVa biliary cysts often coexist with PBM.^[18]^ Further discussions on pathogenesis suggest that biliary cysts with PBM have a higher risk of secondary canceration and should be more clearly distinguished from other Todani types.^[19]^ In this case, the patient had cystic dilation of the extrahepatic common bile duct, and follow-up revealed continuous dilation extending to the left intrahepatic bile duct. Based on the above discussion, the diagnosis is consistent with biliary cyst (Todani Type Ic). The presence of common bile duct stones is considered a long-term complication of PBM.

Based on current evidence-based practice, surgical intervention is the preferred treatment option for biliary cysts complicated by PBM to achieve the goals of preventing carcinogenesis, removing the lesion, and achieving biliary-pancreatic diversion.^[17]^ In this case, initial ERCP biliary stent drainage in 2020 was implemented as a temporary strategy. For early chronic pancreatitis complicated by pancreatic duct protein plugs/stones, ERCP-guided removal of pancreatic duct stones and placement of accessory pancreatic duct stents are effective treatment strategies.^[16]^ After multiple ERCP treatments, the patient ultimately opted for surgical intervention in 2021 and achieved long-term symptom relief. During follow-up in April 2024, a cyst with stones was detected in the lower segment of the common bile duct, which we suspected was due to the unresected pancreatic segment of the bile duct during the previous surgery, which had re-expanded and formed stones under the influence of pancreatic juice reflux. Given reports of secondary carcinogenesis in the residual pancreatic segment of the common bile duct in patients with PBM,^[20]^ this patient still requires close follow-up and may require additional surgery for dissection if necessary.

Post-surgery, the patient experienced relief from abdominal pain, and follow-up revealed no recurrence of local dilation or stones in the pancreatic duct, indicating that the surgery successfully resolved the impact of bile on the pancreatic duct. This suggests that the patient’s early chronic pancreatitis received causative treatment and did not progress further.

## 4. Conclusion

EUS is effective for the diagnoses of complex PBM and incomplete pancreatic divisum. ERCP with pancreatic duct stent placement and surgical procedure is reliable for relieving patient’s symptoms.

## Author contributions

**Investigation:** Yang Lv.

**Resources:** Wei Gong.

**Supervision:** Xiaobing Cui.

**Visualization:** Yingchun Zhang.

**Writing – original draft:** Wen Xu.

**Writing – review & editing:** Ying Zhu.
